# Pus in Spinal Needle: Diagnosis and Management of a Long-Segment Spinal Epidural Abscess

**DOI:** 10.1155/2021/9989847

**Published:** 2021-04-28

**Authors:** B. M. Munasinghe, N. Pathirage, M. S. Hameed, C. T. Hapuarachchi

**Affiliations:** ^1^Department of Anaesthesia and Intensive Care, District General Hospital, Mannar, Sri Lanka; ^2^Consultant Orthopaedic Surgeon, District General Hospital, Mannar, Sri Lanka; ^3^Department of Microbiology, District General Hospital, Mannar, Sri Lanka

## Abstract

Spinal-epidural abscess (SEA) is believed to be primarily of haematogenous origin and very rarely as a consequence of central neuraxial blockade. Early diagnosis and pertinent management invariably improve neurological outcomes. We report a case of long-segment SEA, which was suspected during subarachnoid anaesthesia, subsequently diagnosed and managed appropriately, averting irreversible neurological deficits.

## 1. Introduction

The epidural space contains fat, venous plexus, and small arteries. Arteriolar and venous theories have been proposed in view of explaining the origins of epidural abscesses. [1] As the dura is tightly attached to vertebral bodies and ligaments anteriorly, epidural abscess tends to occur more in the posterior epidural space. The thoracic region being the longest of all three has the most reported cases of epidural abscesses [2]. Considering the cervical-sacral continuity of the epidural space, infection in a single space could spread to multiple levels, with an average of three spinal segments [3]. It is estimated that, around 0.001% epidural catheterizations are complicated by epidural abscesses [4]. Presentation of epidural abscesses can vary from nonspecific symptoms to neurological deficits though absent symptoms merely do not exclude the diagnosis. Multiple risk factors have been implicated. A majority of the clinicians still prefer surgery with a prolonged antibiotic therapy. If left untreated, this could lead to irreversible neurological impairment or even death. Herein, we present a case of a long-segment spinal-epidural abscess (SEA) in an elderly South Asian lady who had trivial symptoms, fortunately diagnosed and managed promptly, precluding long-term neurological morbidity. Interestingly, SEA presenting itself during subarachnoid anaesthesia has never been reported to the best of our knowledge.

## 2. Case Description

**Figure 1:**
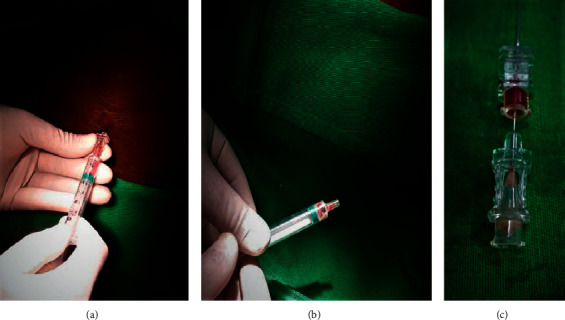
(a)–(c) Pus noted during subarachnoid anaesthesia.

A 67-year-old South Asian female, who was diagnosed with diabetes mellitus with peripheral neuropathy and hypertension, was admitted with right lower limb necrotizing fasciitis to the surgical unit of our institution. A thorough wound debridement was performed under subarachnoid block. Wound swab cultures yielded methicillin-resistant *Staphylococcus aureus* (MRSA) sensitive to teicoplanin. Concomitant blood and urine cultures and rest of the MRSA screening were negative. As she had developed severe reactions to clindamycin, teicoplanin, and penicillin group antibiotics, desensitization for meropenem was carried out in a tertiary-care center under the guidance of a consultant immunologist. Simultaneously, several wound debridements were carried out under spinal anaesthesia. The patient was transferred back to our institution for further wound care. She was on a urinary catheter and utilizing a wheel chair for mobility. A repeat wound debridement was planned under spinal anaesthesia. She had mild back pain with no spinal tenderness or new onset or worsening neurological deficits. History did not reveal any trauma to the back. Throughout her hospital stay, the clotting profile was normal with normal platelet counts, and spinal anaesthesia was conducted under strict aseptic conditions at all instances. While performing the most recent subarachnoid block, the anaesthetist noticed the absence of cerebrospinal flow, and when the spinal needle was withdrawn, pus was noted at the hub (Figures 1(a)–1(c)).

**Figure 2:**
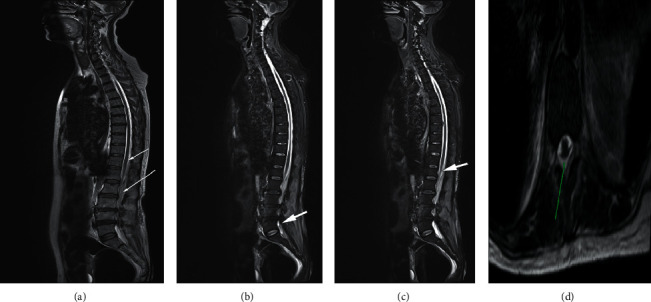
T2-weighted MRI of the spine from the cervical to lumbosacral region depicting a long-segment epidural abscess extending from T11-T12 to L5-S1. Size: 0.8 cm (AP diameter) × 22.5 cm (length) × 1.79 cm (width). No pre- or paravertebral abscesses were visible. (a)-(c): sagittal views. (a): short white arrow- abscess seen commencing at the T12 level and long arrow- abscess extending downwards. (b) and (c): white arrows- abscess visible at L5 segment and the abscess causing cauda equina compression at the L1 level, respectively. (d): axial view; green arrow- abscess at the L4 level.

The procedure was abandoned. With the suspicion of SEA, she was transferred to the nearest neurosurgical center. An urgent MRI of the spine revealed a large epidural abscess with cauda equina displacement and compression with edema involving cauda equina fibres (Figure 2).

Vertebral osteomyelitis and discitis were excluded. There was no evidence of psoas abscess or intra-abdominal collections. Urgent bilateral L4 laminectomy with epidural abscess evacuation was carried out under general anaesthesia. The abscess aspirate grew methicillin-sensitive *Staphylococcus aureus* which was treated with intravenous meropenem for 28 days. Even though the sensitivity for cloxacillin and ceftriaxone was noted in the aspirate, the recent history of severe reactions to penicillin group antibiotics, the cross reactivity between third-generation cephalosporin (ceftriaxone in our patient) and penicillin groups, and her desensitized state for meropenem prompted preference of the latter. She made a complete recovery without general or neurological complications and was discharged 6 weeks later. The routine follow-up in the surgical clinic excluded any recurrence of the SEA.

## 3. Discussion

Generally being a sterile confined region, the epidural space could host a suppurative infection when bacteria enter and seed. It is believed that a majority of the SEA are caused by haematogenous spread [5]. Spread of nearby infections such as vertebral osteomyelitis, discitis or spondylitis, or paraspinal abscesses have also being implicated. More and more spinal surgeries and epidural catheters had contributed to the rising trend of SEA during recent years [6] even though the overall incidence due to the latter is minor. In one-third of cases, no identifiable nidus of infection had been identified [7]. Diabetes mellitus is considered as the commonest predisposing factor for SEA, which could have been the case in our patient [8]. Immunosuppression due to chronic alcoholism, long-term steroid therapy, malignancy, or organ transplantation are other precipitants frequented with SEA. Intravenous drug abuse is identified as a causality in increasing instances at present [9]. Irrespective of the aetiology, *Staphylococcus aureus* has been attributed commonly [8].

Diagnosis of SEA could be challenging due to a variety of factors. Clinically, presentation could be with nonspecific symptoms and symptoms and signs might overlap with the underlying primary aetiology. Importantly, patients with chronic illnesses, spinal pain, and neurological sequelae might go unnoticed. In the case of our patient, who had diabetic peripheral neuropathy with a urinary catheter in situ and immobility due to the lower limb wound, back pain which was of mild intensity was the only attributable feature of an SEA.

In a suspected patient with SEA, MRI with gadolinium is the gold-standard imaging [2]. Although CT with intravenous contrast is less sensitive, it is used as an alternative when the former is not available. The place of lumbar puncture in diagnosis of SEA is discouraged due to the risks of introducing the infection to the subarachnoid space and provision of clinically unremarkable Gram stain or culture patterns [6]. Conversely, the attempted subarachnoid anaesthesia in our patient proved to be crucial, suggesting a sinister pathology and warranting urgent course of action.

The management of SEA depends on the neurological deficit at the time of diagnosis. Surgical evacuation and antibiotic therapy still is the preferred modality of treatment in a vast majority of patients with demonstrable impaired neurology [1, 10]. But, caution with close monitoring is warranted when conservative management is opted, as worsening of neurology could occur with time. A multidisciplinary approach with surgical, radiological, and microbiological input would lead to improved, patient-based management protocols. In this particular patient, multiple drug allergies posed an additional obstacle to the management, but the inclusion of an immunologist was invariably advantageous.

The antibiotic therapy in SEA is generally parenteral and protracted. Duration varies from 4 to 12 weeks [2]. Follow-up is a necessity as recurrences of SEA are not infrequent; however, provided the clinical and basic investigations of infection are normalized, serial radiological tests are usually not required [1].

The mortality and morbidity following SEA are still significantly high [11]. High index of suspicion, relatively low-threshold for diagnostic tests, particularly for patients with multiple risk factors and suggestive clinical findings, a multidisciplinary team approach, and appropriate antibiotics could make the outcome following SEA more favourable and acceptable in this era of sophisticated imaging and antibiotics.

## 4. Conclusions

Atypical presentations of long-segment epidural abscesses should not divert attention from the former in the absence of typical features. SEA attributed to anaesthesia could occur due to infected haematoma following central neuraxial blocks (commonly epidural) or introduction of organisms through unsterile techniques. Conservative or surgical intervention with an extended course of broad-spectrum intravenous antibiotics is indicated accordingly. Presence of vague symptoms in a patient similar to ours beckons a higher degree of suspicion with regard to rarer but devastating complications such as SEA. Prompt multidisciplinary management would lead to better outcomes.

## Data Availability

The authors confirm that the data supporting the findings of this study are available within the article.
