# Corrigendum to “Pus in Spinal Needle: Diagnosis and Management of a Long-Segment Spinal Epidural Abscess”

**DOI:** 10.1155/2021/9893619

**Published:** 2021-08-11

**Authors:** B. M. Munasinghe, N. Pathirage, M. S. Hameed, C. T. Hapuarachchi

**Affiliations:** ^1^Department of Anaesthesia and Intensive Care, District General Hospital, Mannar, Sri Lanka; ^2^Consultant Orthopaedic Surgeon, District General Hospital, Mannar, Sri Lanka; ^3^Department of Microbiology, District General Hospital, Mannar, Sri Lanka

In the article titled “Pus in Spinal Needle: Diagnosis and Management of a Long-Segment Spinal Epidural Abscess” [1], the authors identified an error in the Introduction section. Following the publication of the article, the authors identified a previously published similar case of a patient with an epidural abscess suspected during spinal anaesthesia. The article should therefore be corrected as follows, with the addition of reference 12 [2]:

“Interestingly, SEA presenting itself during subarachnoid anaesthesia has never been reported to the best of our knowledge.” should be corrected to “Interestingly, SEA presenting itself during subarachnoid anaesthesia has very rarely been reported in literature [12].”
